# Prognositic significance of P-cadherin expression in breast cancer: Protocol for a meta-analysis

**DOI:** 10.1097/MD.0000000000014924

**Published:** 2019-03-22

**Authors:** Yupeng Xi, Xiwen Zhang, Zizhen Yang, Xing Zhang, Qiujun Guo, Zhenhua Zhang, Shuntai Chen, Honggang Zheng, Baojin Hua

**Affiliations:** a Beijing University of Chinese Medicine; b Department of Oncology, Guang’anmen Hospital, China Academy of Chinese Medical Sciences, Beijing; c Shanxi University of Chinese Medicine, Xianyang, Shanxi Province, China.

**Keywords:** breast cancer, meta-analysis, P-cadherin, prognosis, protocol

## Abstract

**Background:**

P-cadherin is a calcium-dependent cell–cell adhesion glycoprotein. It has been implicated in invasiveness and metastasis. However, the clinical prognostic value of overexpression of P-cadherin in patients with breast cancer (BC) remains unsettled.

**Methods:**

A systematic literature search will be performed in all available databases to quantitatively review eligible studies and identify all relevant data, which could be used to detect the relationship between overexpression of P-cadherin and overall survival (OS), disease-free survival (DFS), and clinicopathological parameters. Hazard ratio and 95% confidence intervals (CIs) or *P* value will be employed as effect measures to estimate the correlation between P-cadherin and the oncologic outcomes including overall survival (OS), disease-free survival (DFS). Odds ratios (ORs) and the 95% CIs will be evaluated for the pooled analysis of the correlation between P-cadherin expression and clinicopathological features. We will use the Review Manager (Revman) 5.3.5 software (Cochrane Community, London, United Kingdom) and STATA 14 software (version 14.0; Stata Corp, College Station, TX) to perform the meta-analysis to calculate the data.

**Results:**

The review will provide a high-quality synthesis of current evidence of the prognostic role of P-cadherin in BCs. The results will be published in a peer-reviewed journal.

**Conclusion:**

We hope that the results of this study will provide significant evidence to assess whether the expression of P-cadherin is associated with poor prognosis in patients with BC.

**PROSPERO registration number:**

This meta-analysis protocol has been registered in the PROSPERO network with registration number: CRD42019119880.

## 1 Introduction

Breast cancer (BC) is one of the most common malignancies and a leading cause of cancer-related mortality among women worldwide.^[1,2]^ BC can be divided into 4 molecular sub-types^[3]^: Luminal A, Luminal B, HER-2 positive, and triple-negative, based on the expressions of the hormone receptors (estrogen receptor [ER] and progesterone receptor [PR]), human epidermal growth factor receptor-2 (HER-2), and ki-67 of BC tissues. These were applied by oncologists in the outcome prediction and management of BC patients by using different therapeutic regimens, such as chemotherapy, endocrinotherapy, and HER-2 targeted therapy.^[4]^ However, as patients with histologically similar tumors at the same sub-types and clinical stage may have different clinical outcomes,^[5]^ it remains difficult to predict patient survival. So, it need us to precisely diagnose and focus on identifying which therapies are most effective for each patient based on genetic characterization of the cancer.^[6]^ These personalized treatment regimens require a precise molecular prediction of individual disease outcome.^[7]^

Besides the luminal molecular sub-types, researchers have been identifying prognostic biomarkers in tumor patients. Proteins such as epidermal growth factor receptor (EGFR),^[8]^ breast cancer type 1 susceptibility protein,^[9]^ B-cell lymphoma 2,^[10]^ and pro-apoptotic protein p53^[11]^ were found to be associated with the overall survival time of BC. However, it is still necessary to explore established markers possessing predicative values for the survival of BC patients.

P-cadherin, also known as placental cadherin, was originally found in the placental tissue of mice, and its encoding gene, CDH3, is located on chromosome 16th 16q22. 1. Cadherin is a class of Ca^2+^ dependent surface membrane glycoprotein, which is characterized by cell type specific expression pattern, which mediates homotypic cell–cell adhesion and forms 2 dimers. According to the tissue distribution, classic cadherin is mainly divided into CDH1/E-cadherin (epithelial cells), CDH2/N-cadherin (neurons), CDH3/P-cadherin (placenta), and CDH4/R-cadherin (retina). The structure between the members of the family with homology to P-cadherin by 3 different domains: extracellular domain, 5 cadherin repeats (EC1–EC5); transmembrane domain; intracellular domain, with alpha, beta, gamma, P120-Catenin composed of E-cadherin–catenin complex. The cadherin–catenin complex can be linked to the actin cytoskeleton through α-catenin, which plays an important role in the migration of tumor cells. β-catenin is not only involved in the formation of cadherin–catenin complex, but also a key factor regulating the transcriptional activity in WNT/β-catenin signaling pathway, which is related to tumor cells proliferation and differentiation. P120-catenin is regulated by tyrosine kinase, which is related to the stability of cadherin in the cell membrane, regulating the adhesion, and cell movement ability of cadherin and its transport in cells.

P-cadherin is expressed in many malignant tumors. Its mechanism may be related to P-cadherin mediated signal transduction pathway, P-cadherin soluble form, and matrix metalloproteinase (MMPs) secretion. However, the role of P-cadherin in the process of cancer is not clear, and the role in many models is different. For example, P-cadherin, as a tumor suppressor gene in malignant melanoma, can inhibit the invasion and metastasis of the tumor. However, in other tumor models, especially breast cancer, P-cadherin can enhance the invasion and aggression of tumor cells. Therefore, the role of P-cadherin in the diagnosis, prognosis, and treatment of the tumor still needs further validation. Given that the clinical results are controversial, we aim to systematically evaluate the prognostic role of P-cadherin in breast cancer patients.

## 2 Outcomes

The primary outcomes of this review are overall survival (OS), disease-free survival (DFS). The second outcomes are clinicopathological parameters in breast cancer including the following items: histological grade; lymph node metastasis; The TNM classification of malignant tumors stage; the expression of HER-2, EGFR, estrogen receptor, PR, MKI67, cytokeratin 5/6.

## 3 Materials and methods

### 3.1 Standards

The protocol of the meta-analysis will be developed according to the Preferred Reporting Items for Systematic Reviews and Meta-Analysis Protocols (PRISMA-P) statement guidelines.^[12]^ The PRISMA-P checklist of the protocol is supplied in PRISMA-P-checklist.

### 3.2 Ethical issues

Ethical approval is not required because this is a literature-based study based on previously aggregate data and will be no direct contact with individual patients.

### 3.3 Registration

Our meta-analysis protocol has been registered in the PROSPERO network with registration number: CRD42019119880.

### 3.4 Data sources and search strategy

Studies will be searched for in PubMed, EMBASE, Cochrane Library, and CNKI without language limitations. The search time was from January 1997 to October 2018. In addition to electronic databases, printed journals, and relevant textbooks will be manual searched in the libraries of the Beijing University of Chinese Medicine, Peking Union Medical College, and Guang’anmen Hospital. Furthermore, the reference lists of the identified articles obtained from the original search will be manually searched to identify additional relevant studies.

Articles will be qualified using the following combined keywords: “Breast Neoplasm(s),” “Breast Tumor(s),” “Mammary Cancer(s),” “Breast Carcinomas,” “P-cadherin,” “CDH3,” “Cadherin 3,” “P-CAD,” “prognostic,” and “survival,” “outcome,” “prognostic biomarker(s).” The preliminary search strategy in Table 1 will be used for PubMed. This search strategy will be modified and adapted to other databases based on their specific requirements.

**Table 1:**
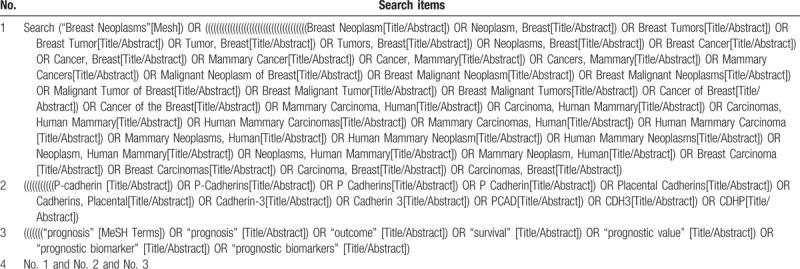
Preliminary search strategy for PubMed.

### 3.5 Inclusion and exclusion criteria

Articles will be included if they meet the following criteria: female patients with histologic diagnosis of breast cancer; P-cadherin protein in the primary tumor tissues can be assessed by using an immunohistochemical method; hazard ratios (HRs) and 95% confidence intervals (CIs) of the statistical results can be extract directly or calculated from demographic data or survival curves, and independent P-cadherin expression level data.

Articles will be excluded if they meet the following criteria: reviews, letters, conference abstracts, comments, and single case reports; studies not focusing on the role of the P-cadherin expression on prognosis or the clinicopathological features in BC.

### 3.6 Studies selection, data extraction, and management

**Figure 1:**
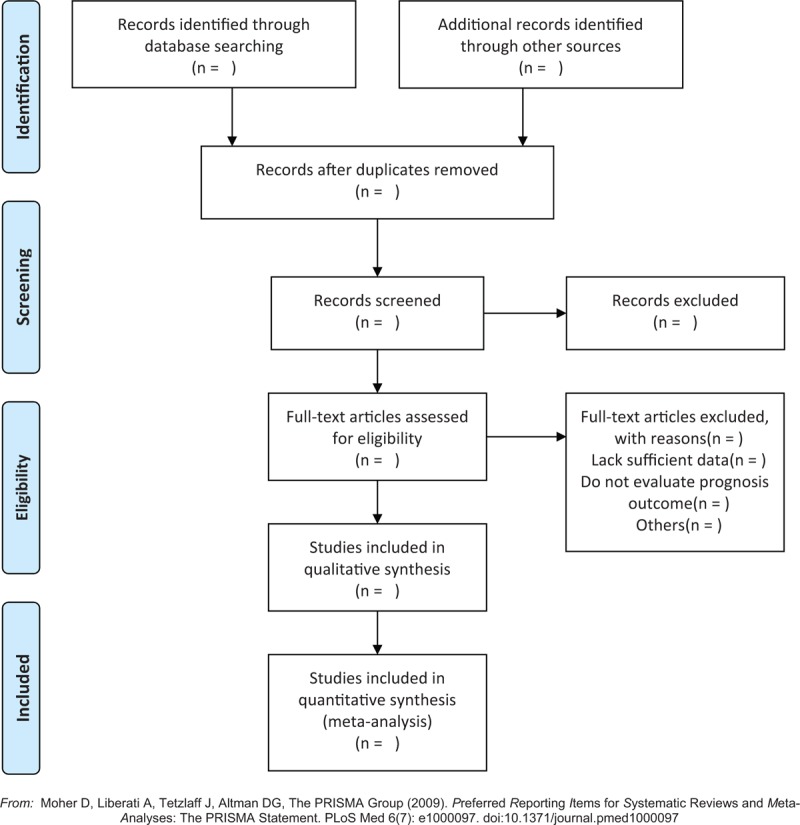
Flow diagram of studies search and selection.

Two reviewers will extract data independently from the eligible studies by using a standardized collection form. The selection process will be summarized according to PRISMA flow diagram (Fig. 1). We will record the details of the eligible studies including first author, characteristics of patients, publication year, pathology type, P-cadherin assay methods, total cases, clinical pathological features, and outcomes. The hazard ratio will be extracted directly if it is reported in the article or it is estimated from the Kaplan–Meier survival curve and the 5-year survival outcome events using the methods reported by Tierney et al.^[13]^ If there are discrepancies between the 2 reviewers, a final consensus will be reached by discussion or an arbiter.

### 3.7 Assessment of quality in included studies

The quality of each selected studies will be independently assessed by 2 investigators using the Newcastle-Ottawa Quality Assessment Scale (NOS) which has been recognized as a validated quality assessment instrument for nonrandomized trials that assesses 3 parameters of study quality: selection, comparability, and outcome assessment.^[14]^ Each study with at least scores 5 is considered as high quality. Discrepancy of quality assessment among the investigators will be resolved through discussion or reached the consensus by an arbiter.

### 3.8 Measures of prognosis and clinicopathological features

In this meta-analysis, HRs with 95% CIs or *P* values will be applied as appropriate values to measure the P-cadherin expression on prognosis (OS and/or DFS) in breast cancer. Odds ratios (ORs) and the 95% CIs will be evaluated for the pooled analysis of the correlation between P-cadherin expression and clinicopathological features.

### 3.9 Assessment of heterogeneity

The heterogeneity of the included studies will be evaluated by the chi-squared-based *Q* test and *I*^2^ test, and *P* < .10 or *I*^2^ > 50% will be defined as indicating heterogeneity.^[15]^

### 3.10 Assessment of publication biases

The publication bias and meta-regression will be evaluated by the Egger test and Begger^[16]^ test with *P* < .05 indicating significant bias and a contribution of heterogeneity.

### 3.11 Statistical analysis

The statistical analyses will be performed using Review Manager (Revman) 5.3.5 software (Cochrane Community, London, United Kingdom) and STATA 14 software (version 14.0; Stata Corp, College Station, TX). The value of HR and the 95% CI describing OS and/or DFS will be obtained directly in some studies. In addition, many studies displaying *P*-values of the log-rank test or Kaplan–Meier survival curve will be inferred using Tierney method. The dichotomous data of the clinical pathological features will be pooled using odds ratios (ORs) with 95% CIs. The fixed effect model will be used for pooling homogeneous data^[17]^ (*I*^2^ < 50% or *P* > .10) and the random effect model will be used for heterogeneous data^[18]^ (*I*^2^ > 50% or *P* < .10). In cases of substantial heterogeneity, the source of the heterogeneity will be detected by the subgroup analysis and sensitivity analysis.

### 3.12 Subgroup analysis

Subgroup analysis will be detected to exploring the causes of heterogeneity. Different countries and different pathological types of breast cancer will be considered as subgroup analysis.

### 3.13 Sensitivity analysis

Sensitivity analysis will be performed by reanalyzing the data using different statistical methods, mainly including methods that use sequential omitting individual studies to test the stability of the pooled results.

## 4 Discussion

Breast cancer is among the most deadly malignancies in developed countries, with metastatic spread being the major reason behind this fatal outcome.^[19]^ It is noteworthy that, so far, a considerable number of investigations have been completed on the relationship between P-cadherin levels and the prognosis of BC patients. Some studies have shown that P-cadherin is overexpressed in triple-negative breast cancers above 50%, significantly associated with poor patient survival,^[20–22]^ and shows key effects in some acquired cancer hallmarks, such as increased cell migration, invasion, as well as tumorigenic and metastatic capacity in breast cancer models.^[23,24]^ However, the results of these studies are controversial, and no consensus has yet been reached. Hence, we hope this review will provide more accurate and objective evidences of the relationship between the P-cadherin and the prognosis of patients with BC. The findings will be published in a peer-reviewed journal.

However, this review still has some limitations. Due to language barriers, only studies published in Chinese and English will be included. Besides, different countries and histology type of BC may run risk of heterogeneity.

## Author contributions

Yupeng Xi and Qiujun Guo contributed to the conception of the study. Yupeng Xi, Xiwen Zhang, and Honggang Zheng wrote the draft of manuscript, and was revised by Zizhen Yang, Xing Zhang, Zhenhua Zhang, and Shuntai Chen, the search strategy was developed by all of the authors. Yupeng Xi, Xiwen Zhang, and Honggang Zheng will search, extract data, assess the risk of bias, and complete the data synthesis. Baojin Hua will arbitrate in case of disagreement and ensure the absence of errors. All authors approved the publication of the protocol.

**Conceptualization:** Yupeng Xi, Qiujun Guo.

**Data curation:** Yupeng Xi, Xiwen Zhang, Zhenhua Zhang, Shuntai Chen.

**Funding acquisition:** Honggang Zheng, Baojin Hua.

**Methodology:** Xiwen Zhang, Xing Zhang, Shuntai Chen, Honggang Zheng.

**Project administration:** Zizhen Yang, Xing Zhang, Zhenhua Zhang.

**Supervision:** Honggang Zheng, Baojin Hua.

**Validation:** Baojin Hua.

**Writing – original draft:** Yupeng Xi.

**Writing – review & editing:** Xiwen Zhang, Honggang Zheng.
